# Dental Periodical Literature—Dental Journals

**Published:** 1859-12

**Authors:** 


### DENTAL REGISTER OF THE WEST.

Vol. XIII.] DECEMBER, 1859. [No. 6.

### Original Essays and Communications.

### "DENTAL PERIODICAL LITERATURE."—"DENTAL JOURNALS."

"The best laid scheme o' mice an' men
Gang aft agley,"

And sometimes, in spite of the best attention and closest scrutiny of the machinist, a screw gets loose, and the whole machinery is deranged and torn to pieces. Indeed, the whole moral machinery of our race is sadly out of joint, and the physical is but little better. This, however, might be borne with some degree of patience, as we have become pretty well used to it, were it not that, as a consequence, our "periodical literature" falls short of what it ought to be. Sad and sudden is the news! but true it is that there is something wrong with our "dental journals"—with our "dental periodical literature." There must be; for two unimpeached witnesses, examined "separate and apart, and without the hearing of" each other, have, simultaneously, testified substantially to this fact.

The evidence of one of these witnesses is recorded in the editorial department of the "*Cosmos*," that of the other, in the "original" department of the "*American Journal.*" In the latter, it is intimated that our periodical literature was at one time pretty good, at least "from fair to middling," but that it has degenerated in quality, in proportion as it has increased in quantity. Still, according to this witness, it has lost but little of interest, whatever it may have lost in character; for he says, "No subject is more interesting to the observer of the progress of dentistry than the state of its literature, and more particularly, its periodical literature." Still he tells us, "It is difficult to say whether dentists should esteem it or not;" but before he gets through, the difficulty vanishes, and he tells us that it "can not be considered as reflecting credit upon dentists." We thought, at first reading, that these sentiments were slightly contradictory, but were reminded that even a cock-fight "is more interesting to the observer" *sometimes*, than any other "subject;" yet it should not be esteemed, and "can not be considered as reflecting credit upon" those who bring it about.

The other witness seems to intimate that dental journals have always been highly defective, and that a retrospective view of them would call for mortifying acknowledgments and admissions, on account of the "unpardonable laxity" manifested in regard to the admission of "effusions of ignorance, the ebullitions of passion," "the cant phrases of the vulgar," and other "sad trash." They do not agree as to the result of a "retrospective view." One finds in the journals of bygone periods a waste of "*time*, *talents* and *energies*" in "the development of personalities," and, even yet worse, the employment of "expressions that are usually left to the vulgar and illiterate, and which, when found in the pages of a professedly scientific magazine, *excites* in the mind of the gentleman, and devotee of science, a feeling of loathing and disgust." (We would clip the tail of that last verb if it was ours; for, in the expressive language of its proprietor, it "sets all the rules of grammar at defiance.") The other finds the present view bad, but in the retrospective one quite satisfactory, and endeavors to have the present transformed into the same image. Accordingly, he appeals to the men of education and dignity to stand forward, and *renew* their efforts with the same zeal which actuated them in the beginning. He regards the present "profession" as a band of "sutlers," as "hawkers and criers," and charges the true profession, "with the exception of two or three of the greatest names," (himself and us, of course,) with the cowardly crime of retiring before them. He can't excuse these retired "veterans" on account of age; for, he maintains that "fifteen or twenty years has surely not made old and unqualified men of those who were then youthful." And if they "has" not, his doctrine, and a correct one, is that they must go to work again, or "the contempt of the future awaits them." But as these men were "youthful" when they wrote so gloriously for our periodicals, we hope the *youthful* of the present day will take courage from, and emulate their example, in all save their inglorious retirement; for we believe that the youthful men of to-day are at least as talented as the youthful men of any former period.

But the reader must not despond. The condition of our periodical literature is not hopeless; for we are told that, "out of the four or five thousand dentists in this country, a sufficient number might be found to furnish one or two periodicals with the proper material." And we are still more encouraged when we are told that these efficient individuals "have lately been found." Now, if "the able-minded deserters should again be brought into the ranks to serve out their term of enlistment," we will have a "revival of letters" in our periodical literature. It is consoling, too, to learn that we are not worse than, if even as bad as our neighbors. The British Journals are condemned as imperfect too; but we are told that they "should not be judged harshly," as they "are yet *in embryo*." This is certainly considerate; but we would suggest greater mildness still. Unborn babes should not be judged at all, especially when "yet *in embryo*." Why, then, should *embryonic* periodicals be held accountable? In the name of "unborn millions," we protest against such injustice. The English journals, however, are advised to use "the knife;"

and the writer adds, "In England, Americans are noted for using knives, therefore, our present advice will not suggest thoughts of a Franco-American alliance." But does it not rather suggest a resort to the Cesarean operation, at too early a period of utero-gestation, as the periodicals are still "*in embryo.*" But this can hardly be the intention of the writer; for he tells us that he means "more properly the pruning shears, as we look at them from a gardener's point of view. They are the semi-respectable fruits of our toil." We don't know the meaning of this sentence; but if the writer intends to say that pruning shears are only *semi-respectable*, we differ with him.

But we believe the two articles under consideration, the one in the October number of the "*Cosmos*," and the other in the "*American Journal*" of the same date, will do great good. The one urges the "deserters" to renew their work, because the present force is insufficient; the other as strongly urges the present force to work better than their predecessors, and we heartily sympathize with both. This sympathy, however, will not induce us to join them in singing their Jeremiads over the follies and failings of our dental periodicals; for though they are far from perfect, yet we are not ashamed of them. They will compare very favorably with the periodicals of any other profession; and there has been no period of their existence that they would not.

The writer in the "*Cosmos*" gives his "*senior*" some very good advice as to the management of his department of the journal, which was, we think, unnecessary, yet it was highly consistent that he should give it; for a man, during the first year of his editorial labors, is wiser (Proverbs, xxvi, 16) than at any subsequent period. This must not be disputed; for we speak "*by experience.*"

But lest our friend should be too disconsolate on account of the admission into the journals, of papers "faulty in language," "defective in structure," containing "inelegant lan. guage," and not "well written," in short, lest he should pine too sadly for essays as painfully accurate as the school-girl compositions of Miss Araminta Snooks, we would suggest, as a balm to his troubled breast, a few remarks from a criticism in the "*Atlantic Monthly.*"

In speaking of the effect of our public schools and the universal reading of newspapers, this critic says, "This has tended to make the common language of talk more bookish, and has thus reacted unfavorably on our literature, giving it sometimes the air of being composed in a dead tongue, rather than written from a living one." And again,

"A dialect that is taught grows more and more pedantic, and becomes at last as unfit a vehicle for living thought as monkish Latin. This is the danger which our literature has to guard against from the universal schoolmaster, who wars upon home-bred phrases, and enslaves the mind and memory of his victims, as far as may be, to the best models of English composition,—that is to say, to the writers whose style is faultlessly correct, but has no blood in it."

For our own part, we prefer a "blooded" style to "a potted literature;" and we believe that our taste is not singular.

